# Serotonin Exhibits Accelerated Bleomycin-Induced Pulmonary Fibrosis through TPH1 Knockout Mouse Experiments

**DOI:** 10.1155/2018/7967868

**Published:** 2018-04-16

**Authors:** Jingyao Zhang, Ruixia Cui, Yang Feng, Weiman Gao, Jianbin Bi, Zeyu Li, Chang Liu

**Affiliations:** ^1^Department of Hepatobiliary Surgery, The First Affiliated Hospital of Xi'an Jiaotong University, Xi'an, Shaanxi 710061, China; ^2^Department of Surgical Intensive Care Unit, The First Affiliated Hospital of Xi'an Jiaotong University, Xi'an, Shaanxi 710061, China; ^3^Department of ICU, The First Affiliated Hospital of Xi'an Jiaotong University, Xi'an, Shaanxi 710061, China; ^4^Department of Immunology, Shaanxi University of Chinese Medicine, Xianyang, Shaanxi 712046, China; ^5^Xi'an Jiaotong University Health Science Center, Xi'an, Shaanxi 710061, China

## Abstract

**Background:**

Pulmonary fibrosis is a chronic progressive fibrosis interstitial lung disease that is characterized by inflammatory infiltration and fibrotic changes. 5-Hydroxytryptamine (5-HT) is an important regulatory factor in inflammation, immunomodulation, and fibrosis. The aim of this study was to investigate the role of 5-HT in bleomycin- (BLM-) induced pulmonary fibrosis through wild-type C57BL/6 (WT) and TPH1 knockout (KO) mouse experiments.

**Methods:**

The mice were grouped as follows: WT control group, KO control group, WT BLM group, and KO BLM group. Mice were administrated bleomycin hydrochloride through intratracheal instillation to induce pulmonary fibrosis. Mice were sacrificed 0, 7, 14, and 21 days after modeling, and bronchoalveolar lavage fluid (BALF) and lung tissues were collected to determine the severity of fibrotic changes.

**Results:**

The results showed that the weight loss of mice in the WT BLM group was more severe than that in the KO BLM group. H&E and Sirius Red staining revealed that 5-HT markedly aggravated histological damage and fibrotic changes in the lung. Significantly lower levels of hydroxyproline, Ashcroft fibrosis score, total BALF protein and cells, BALF tumor necrosis factor- (TNF-) *α* and interleukin- (IL-) 6, TNF-*α* and IL-6 mRNA, malondialdehyde (MDA), and myeloperoxidase- (MPO-) positive cells in the lung tissues, and fibrosis-associated proteins were discovered in the mice from the KO BLM group compared with the WT BLM group.

**Conclusion:**

5-HT aggravated pulmonary fibrosis mainly by promoting the inflammation, exudation of proteins and cells, oxidative stress, and upregulation of fibrosis-associated genes in the lung tissues.

## 1. Introduction

Idiopathic pulmonary fibrosis (IPF) is a type of progressive and irreversible chronic fibrotic interstitial lung disease with only 2–3-year survival once diagnosed [1]. Progressive dyspnea and a dry irritating cough are the major clinical presentations [2]. Although the exact molecular mechanisms that cause IPF progression are not fully illustrated, the commonly accepted pathogenesis involves inflammatory injury to the alveolar epithelium, excessive extracellular matrix (EMC) deposition, persistent proliferation and accumulation of fibroblasts, abnormal repair and remodeling of lung tissue, and so on [3, 4]. The available medical therapeutic options for IPF are poorly effective. Corticosteroids and anti-inflammatory, antioxidative stress, immunomodulatory agents, as well as antifibrotic agents, only have partial effects but fail to produce invertible benefits [5–7]. Thus, exploring the precise mechanisms and designing relative targeted drugs are urgent for the conquest of IPF.

Serotonin, also known as 5-hydroxytryptamine (5-HT), is a small monoamine molecule derived from tryptophan. The central 5-HT synthesized by tryptophan hydroxylase 2 (TPH 2) is involved in the regulation of cognition, mood, aggression, mating, feeding, and sleep. The peripheral 5-HT synthesized by TPH1 regulates platelet aggregation, bone development, immune responses, and inflammatory reaction [8, 9]. Previous studies indicated that serotonin played essential roles in fibrosis lesions, including adventitia fibrosis during the pulmonary arterial hypertension process, liver fibrosis, dermal fibrosis, and postoperative intra-abdominal adhesion (PPA) formation [10–13]. However, few studies have looked at the role of 5-HT in pulmonary fibrosis, while indirect evidence showed that elevated 5-HT levels in serum, bronchoalveolar lavage fluid (BALF), and lung homogenates could be observed in pulmonary fibrosis mice [14, 15]. This study used TPH1 knockout (deficiency of peripheral 5-HT) and wild-type C57BL/6 (sufficiency of peripheral 5-HT) mice to investigate the role of 5-HT in bleomycin-induced pulmonary fibrosis, which may allow 5-HT as a biomarker of early detection of pulmonary fibrosis and a potential therapy target.

## 2. Materials and Methods

### 2.1. Experimental Animals

The study was conducted by using male TPH1 knockout (KO) mice and male wild-type C57BL/6 (WT) mice (4–5 weeks old, weighing 20–25 g) (Animal Feeding Center of Xi'an Jiaotong University Health Science Center). All the mice were housed in a specific pathogen-free facility with a standard animal diet and water ad libitum. All animal procedures were reviewed, approved, and supervised by the Institutional Animal Care and Use Committee of the Ethics Committee of Xi'an Jiaotong University Health Science Center, China. The bleomycin was purchased from Sigma-Aldrich (Saint Louis, MO, USA).

### 2.2. Study Design

The prepared mice were anesthetized by intramuscular injection of chloral hydrate (10 mg/kg, 4%). Then, the mice were treated with a cannula inserted into the trachea and down into the lungs. A drug was slowly instilled into the lungs by this cannula. The mice were grouped as follows: (1) WT control group, (2) KO control group, (3) WT BLM group, and (4) KO BLM group. Intratracheal instillation of bleomycin hydrochloride (2 UI/kg, dissolved in 0.5 ml 0.9% sterile saline) was performed to induce pulmonary fibrosis in the WT BLM and KO BLM groups. Meanwhile, the mice in WT and KO control groups were treated with an intratracheal instillation of equivalent saline. To ensure sufficient distribution of the drug, 0.5 ml air was injected twice after intratracheal instillation. Six mice per group were used in this study. The body weight of the mice was calculated every day. On days 0, 7, 14, and 21, the animals were sacrificed after being anesthetized with isoflurane gas to determine the fibrotic changes.

### 2.3. Histological Examination and Scoring

After the mice were sacrificed at a specified point in time, the lung tissues were removed, fixed in 10% formalin solution, and embedded in paraffin. Serial sections (5 *μ*m) were obtained and subjected to by hematoxylin and eosin (H&E) and Sirius Red staining to evaluate the lung pathology and fibrotic changes. The morphological results were assessed blindly by two pathologists. The inflammatory score, fibrotic score, and Ashcroft fibrosis score were calculated according to the criteria presented in Tables 1–3.

### 2.4. Hydroxyproline Quantification

Twenty-one days after bleomycin or saline administration, the lung tissues from all groups were collected to determine the hydroxyproline levels. Hydroxyproline levels were determined using hydroxyproline assay kits (Nanjing Jiancheng Bioengineering Institute).

### 2.5. Bronchoalveolar Lavage Fluid (BALF) Analysis

Bronchoalveolar lavage fluid (BALF) was collected by 3 washes with 1 ml 0.9% saline by tracheal intubation in mice. The supernatants of BALF homogenates were collected to detect the total cell counts and proteins as previously described [16].

### 2.6. Enzyme-Linked Immunosorbent Assays (ELISA)

The BALF TNF-*α* and IL-6 levels were detected by using TNF-*α* and IL-6 ELISA kits (Dakewe, Shenzhen, China).

### 2.7. Measurement of Oxidative Stress

The malondialdehyde (MDA), superoxide dismutase (SOD), and glutathione (GSH) levels in the lung tissues were measured using activity assay kits (Nanjing Jiancheng Bioengineering Institute).

### 2.8. RNA Isolation and Quantitative Reverse Transcription Polymerase Chain Reaction (qRT-PCR) Analysis

Total lung tissue RNAs from all groups were isolated by adopting RNAfast200 Kits (Fastagen Biotech, Shanghai, China). PrimeScript RT reagent kits were adopted to perform reverse transcription (TaKaRa Biotechnology, Dalian, China). The mRNA expression was detected in triplicate and standardized by comparison with 18S. The relative levels were calculated using the comparative-Ct method (*Δ*ΔCt method). The primers used in the study were as follows: TNF-*α*: forward 5′-AAGCCTGTAGCCCACGTCGTA-3′ and reverse 5′-AGGTACAACCCATCGGCTGG-3′; IL-6: forward 5′-TCCATCCAGTTGCCTTCTTG-3′ and reverse 5′-TTCCACGATTTCCCAGAGAAC-3′; and 18S: forward 5′-AAACGGCTACCACATCCAAG-3′ and reverse 5′-CCTCCAATGGATCCTCGTTA-3′.

### 2.9. Immunofluorescence Staining

Serial lung sections pretreated with proteinase K were incubated using a monoclonal rabbit anti-MPO antibody (Santa Cruz Biotechnology Inc., CA) diluted 1 : 400 in PBS. Then, stained sections were washed and incubated with an Alexa Fluorophore 488 nm donkey anti-rabbit antibody at 1 : 300 in PBS for 90 min and counterstained with 4′-6-diamidino-2-phenylindole (DAPI). The results were detected using an inverted Leica CTR 6000 fluorescence microscope and Leica Application Suite Advanced Fluorescence software (Leica UK, Milton Keynes).

### 2.10. Western Blotting

Lung proteins were separated by 10% SDS-PAGE electrophoresis and transferred onto nitrocellulose membranes. The membranes were incubated with mouse antibody collagen I, TGF-*β*1, and *β*-actin after blocking with 10% skim milk at room temperature for 3 h. After washing with PBS, the membranes were further incubated with secondary antibody for 1.5 h. Immune-reactive protein bands were detected by the diaminobenzidine method. The relative density of protein expressions was quantitated by ImageJ software (https://imagej.nih.gov/ij/). Protein levels were standardized by comparison with *β*-actin.

### 2.11. Statistical Analysis

All the data were expressed as mean ± SD. The *t*-test or one-way ANOVA was applied to analyze the difference between groups. *P* < 0.05 represented a significant difference. GraphPad Prism software 6.0 (version 6.0, GraphPad Software Inc., La Jolla, CA, USA) was used for data statistics and statistical mapping.

## 3. Results

### 3.1. 5-HT Worsened Body Weight Loss Induced by Bleomycin in Mice

**Figure 1:**
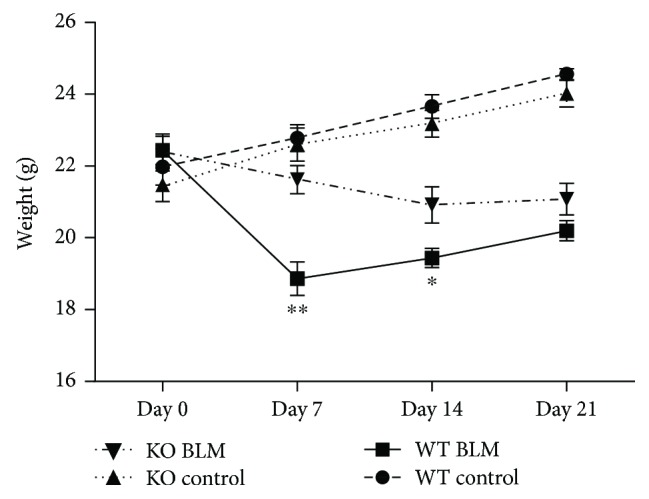
Effect of 5-HT on weight loss induced by bleomycin in mice. Intratracheal instillation of bleomycin (BLM) hydrochloride was performed to induce pulmonary fibrosis in wild-type C57BL/6 (WT) and TPH1 knockout (KO) mice. The changes in body weight of mice from the WT and KO control, WT BLM, and KO BLM groups were examined over the 21-day study period. All data were expressed as mean ± SD, *n* = 6. ^∗^*P* < 0.05 and ^∗∗^*P* < 0.01 versus the WT BLM group.

The weight of mice from the WT and KO control groups increased gently from day 0 to day 21 after the experiment. However, the WT BLM group mice injected with bleomycin showed a significant loss of body weight until day 7 and gradually increased from day 7 to day 21. In contrast, the KO BLM group mice displayed less weight loss, especially during day 0 to day 14. There was a significant decrease in body weight in the WT BLM group mice when compared with the KO BLM group on day 7 (*P* < 0.001) and day 14 (*P* < 0.05), respectively (Figure 1).

### 3.2. 5-HT Aggravated Bleomycin-Induced Pulmonary Fibrosis in Mice

**Figure 2:**
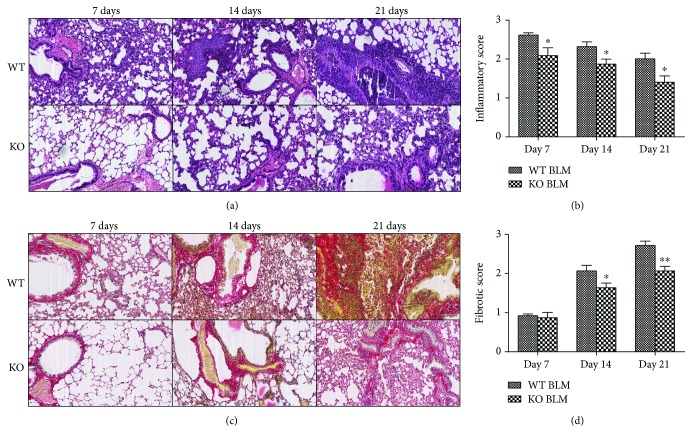
Histological examination of the effect of 5-HT on bleomycin-induced pulmonary fibrosis. Intratracheal instillation of bleomycin (BLM) hydrochloride was performed to induce pulmonary fibrosis in wild-type C57BL/6 (WT) and TPH1 knockout (KO) mice. The mice in the WT BLM and KO BLM groups were sacrificed on days 7, 14, and 21, and histological examination was performed by (a) H&E staining and (c) Sirius staining. The (b) inflammatory score and (d) fibrotic score were calculated to determine the severity of inflammation and fibrosis. All data were expressed as mean ± SD, *n* = 6. ^∗^*P* < 0.05 and ^∗∗^*P* < 0.01 versus the WT BLM group.

Histological examination (H&E staining) of the lungs from both WT BLM and KO BLM groups mice displayed remarkable lung parenchymal fibrotic lesions and inflammatory infiltration when compared with the WT and KO control groups on days 7, 14, and 21 (Figure 2(a)). However, WT BLM group mice were more susceptible to bleomycin toxicity, as evidenced by a higher inflammatory response score (Figure 2(b)). Meanwhile, Sirius Red staining showed that more collagenous fiber formation was observed in the WT BLM group mice on days 14 and 21, which was also confirmed by fibrotic scoring (Figures 2(c) and 2(d)).

### 3.3. 5-HT Promoted Collagen Deposition Induced by Bleomycin in Mice

**Figure 3:**
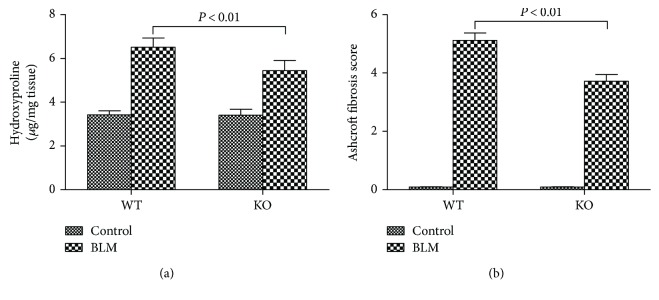
Effect of 5-HT on collagen deposition induced by bleomycin in mice. Intratracheal instillation of bleomycin (BLM) hydrochloride was performed to induce pulmonary fibrosis in wild-type C57BL/6 (WT) and TPH1 knockout (KO) mice. The mice in the WT and KO control, WT BLM, and KO BLM groups were sacrificed on day 21, and lung tissues were collected to determine the (a) hydroxyproline levels and the (b) Ashcroft fibrosis score based on histological examination. All data were expressed as mean ± SD, *n* = 6.

The hydroxyproline level and Ashcroft fibrosis score are positively related to the degree and severity of pulmonary fibrosis [17]. Thus, hydroxyproline levels in lung tissues and Ashcroft fibrosis scores were calculated on day 21 to determine the role of 5-HT in pulmonary fibrosis. As shown in Figure 3(a), mice from the KO BLM group exhibited a significant reduction in hydroxyproline levels compared with the WT BLM group. Meanwhile, WT BLM group mice had a higher Ashcroft fibrosis score, which reflected more severe fibrotic lesions (Figure 3(b)).

### 3.4. 5-HT Increased Exudation of Proteins and Cells Induced by Bleomycin in Mice

**Figure 4:**
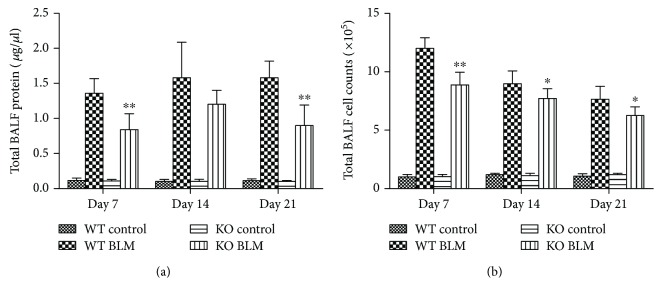
Effect of 5-HT on protein and inflammatory cell infiltrations in BALF induced by bleomycin in mice. Intratracheal instillation of bleomycin (BLM) hydrochloride was performed to induce pulmonary fibrosis in wild-type C57BL/6 (WT) and TPH1 knockout (KO) mice. The mice in the WT and KO control, WT BLM, and KO BLM groups were sacrificed on days 7, 14, and 21, and bronchoalveolar lavage fluids (BALF) were collected to determine the (a) total BALF proteins and (b) total BALF cell counts. All data were expressed as mean ± SD, *n* = 6. ^∗^*P* < 0.05 and ^∗∗^*P* < 0.01 versus the WT BLM group.

The total BALF proteins contain laminin, procollagen 1, procollagen 3, and so on, and the BALF cells mainly include alveolar macrophages, lymphocytes, and neutrophils. They all play important roles in the development and progression of pulmonary fibrosis [18]. Total BALF proteins and cell counts in different experimental groups were examined on days 7, 14, and 21. The total BALF proteins and cell counts in the WT control and KO control groups fluctuated at a fixed value. Total BALF proteins were decreased significantly in KO BLM group mice on days 7 and 21 compared with the WT BLM group (Figure 4(a)). Meanwhile, compared with the KO BLM group, more total BALF cell counts were observed on days 7, 14, and 21 in WT BLM group mice (Figure 4(b)).

### 3.5. 5-HT Increased Inflammatory Reaction Induced by Bleomycin in Mice

**Figure 5:**
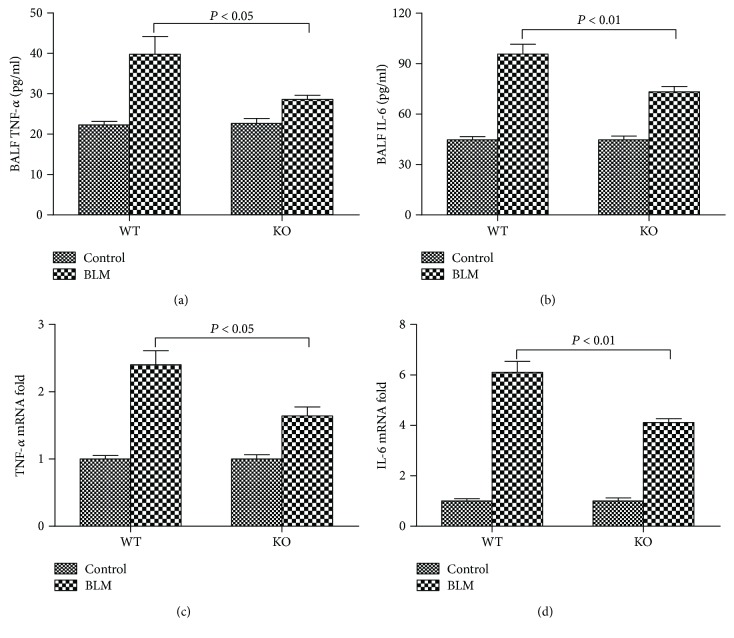
Effect of 5-HT on inflammatory reaction induced by bleomycin in mice. Intratracheal instillation of bleomycin (BLM) hydrochloride was performed to induce pulmonary fibrosis in wild-type C57BL/6 (WT) and TPH1 knockout (KO) mice. The mice in the WT and KO control, WT BLM, and KO BLM groups were sacrificed on day 21. Bronchoalveolar lavage fluids (BALF) were collected to determine the BALF (a) tumor necrosis factor- (TNF-) *α* and (b) interleukin- (IL-) 6 levels. Lung tissues were collected to detect the transcriptional level of (c) TNF-*α* and (d) IL-6 mRNA by quantitative reverse transcription polymerase chain reaction (qRT-PCR) analysis. All data were expressed as mean ± SD, *n* = 6.

BALF TNF-*α* and IL-6 levels were detected to ascertain the severity of inflammation in all groups. Significant increases in BALF TNF-*α* and IL-6 levels were observed 21 days after bleomycin administration in both WT BLM and KO BLM groups mice. However, the KO BLM group mice displayed lower BALF cytokine levels than the WT BLM group mice did (Figures 5(a) and 5(b)). Moreover, we isolated lung RNAs and measured the mRNA levels of TNF-*α* and IL-6 in all groups by qRT-PCR. The results showed that lower transcriptional levels of TNF-*α* and IL-6 were detected in KO BLM group mice in comparison to WT BLM group mice (Figures 5(c) and 5(d)).

### 3.6. 5-HT Increased Neutrophil Infiltration and Oxidative Stress Induced by Bleomycin in Mice

**Figure 6:**
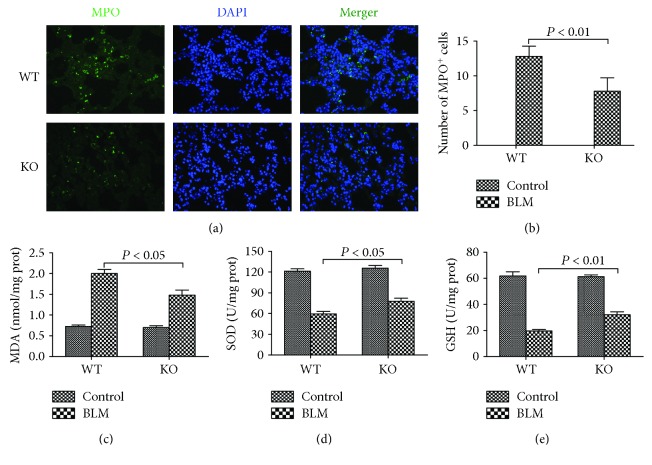
Effect of 5-HT on neutrophil infiltration and oxidative stress induced by bleomycin in mice. Intratracheal instillation of bleomycin (BLM) hydrochloride was performed to induce pulmonary fibrosis in wild-type C57BL/6 (WT) and TPH1 knockout (KO) mice. The mice in the WT and KO control, WT BLM, and KO BLM groups were sacrificed on day 21. Lung tissues were collected to perform immunofluorescent staining to detect the location of (a) myeloperoxidase (MPO) and the calculation of (b) MPO positively stained cells. Meanwhile, the lung (c) malondialdehyde (MDA), (d) superoxide dismutase (SOD), and (e) glutathione (GSH) levels were examined to determine the severity of oxidative stress. All data were expressed as mean ± SD, *n* = 6.

Some evidence suggests that neutrophil infiltration and oxidative stress play significant roles in pulmonary fibrosis [19, 20]. The lung tissues were collected on day 21 to detect these indexes. Immunofluorescence staining of MPO was performed to determine the neutrophil infiltration. The results showed that MPO was mainly distributed in the cytoplasm and subsequently the cell nucleus (green fluorescence) after bleomycin administration. The immunofluorescence staining of MPO was much more positively stained in WT BLM group mice (Figure 6(a)). The quantification of MPO-positive cells showed the same result (Figure 6(b)). The MDA, SOD, and GSH levels in the lung tissues, which reflected the oxidative stress and antioxidant ability, were also measured. Significantly lower MDA levels and higher SOD and GSH levels were observed in the KO BLM group compared with the WT BLM group (Figures 6(c)–6(e)).

### 3.7. 5-HT Promoted Expression Levels of Fibrosis-Related Genes Induced by Bleomycin in Mice

**Figure 7:**
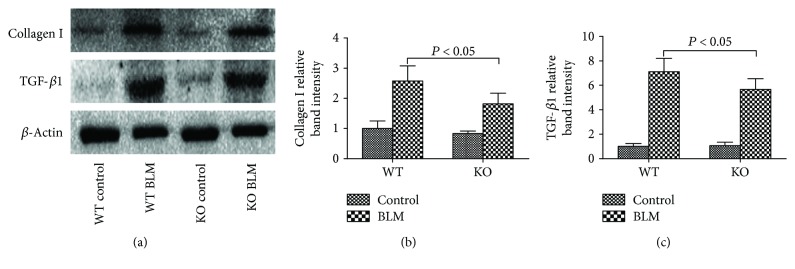
Effect of 5-HT on fibrosis-related gene expression levels induced by bleomycin in mice. The mice in the WT and KO control, WT BLM, and KO BLM groups were sacrificed on day 21. Western blotting was adopted to detect the expression of (a) collagen I and transforming growth factor- (TGF-) *β*1 in the lung tissues. The band intensities of (b) collagen I and (c) TGF-*β*1 were analyzed. All data were expressed as mean ± SD, *n* = 6.

Collagen I and TGF-*β*1 are involved in the development of pulmonary fibrosis and are also the markers of lung remodeling. The lung tissues from all groups were collected on day 21 to detect the expression levels of these fibrosis-related genes. The Western blot results showed that collagen I and TGF-*β*1 were much more expressed in the WT BLM group than in the KO BLM group, which was also confirmed by intensity quantitation (Figures 7(a)–7(c)).

## 4. Discussion

Idiopathic pulmonary fibrosis is a chronic fibrotic interstitial lung disease that is a huge health burden worldwide [21]. Despite the rapid progress achieved in the understanding of pulmonary fibrosis pathogenesis, the precise mechanisms remain unclear. Bleomycin is a classical antineoplastic drug that is also commonly applied for pulmonary fibrosis modeling in rodents. Intratracheal administration of bleomycin directly induces cellular DNA strand break, oxidative stress and inflammation, fibroproliferation, and collagen production [22]. In the present study, we exhibited direct experimental results demonstrating that wild-type C57BL/6 mice (sufficiency of peripheral 5-HT) were more susceptible to bleomycin-induced pulmonary fibrosis compared with TPH1 knockout mice (deficiency of peripheral 5-HT). Lung tissues from WT BLM group mice exhibited more severe histological lesions, collagen deposition, inflammatory reactions, oxidative stress, and higher expression of fibrosis-related genes, which presented direct evidence that 5-HT could aggravate bleomycin-induced pulmonary fibrosis. Indirect clews pointed that serotonin levels in lung homogenates increased significantly during bleomycin-induced pulmonary fibrosis, and blockage of 5-HT2A and 5-HT2B receptors could alleviate the fibrotic changes [14] Pulmonary fibrosis is characterized pathologically by ECM accumulation and pulmonary architecture remodeling. It is associated with unbalanced processes, including proliferation and apoptosis of fibroblasts and accumulation and breakdown of ECM [20]. Although the detailed cellular and modulatory mechanisms of these processes are complicated and still not well illuminated, collagen deposition, inflammation, and oxidative stress are three acknowledged factors evolving pulmonary fibrosis [23, 24]. Collagen deposition is the characteristic pathological change in fibrosis disease. In the study, we found that more positive Sirius Red staining, more hydroxyproline levels, higher fibrotic scores, and Ashcroft fibrosis scores in the lung tissues from WT BLM group mice were observed. All these results indicated that 5-HT could increase collagen deposition in pulmonary fibrosis. In addition to lung fibrosis, the 5-HT system could also stimulate increased hepatic stellate cell proliferation and collagen deposition in liver fibrosis, the production of ECM in dermal fibroblasts, and the production of fibrin formation in PPA formation [11–13]. To study the related molecular mechanisms, we detected the expression of collagen 1 and TGF-*β*1, which are the key proteins in the fibrogenic system. The results showed that 5-HT could significantly increase these protein expressions. TGF-*β*1 is regarded as the most important fibrogenic cytokine that is mainly expressed in fibroblasts, epithelial, and endothelial cells [25]. Preclinical and clinical studies have shown that TGF-*β*1 is significantly upregulated during the progression of fibrotic diseases by stimulating the production of ECM proteins [26, 27]. Chen et al. found that 5-HT could promote adventitia fibrosis through the TGF-*β*1/Smad3 pathway [10]. In our previous study, we also found that 5-HT could promote TGF-*β*1 expression in PPA formation [13].

Neutrophils play an important role in lung parenchyma damage not only in acute lung injury but also in chronic pulmonary fibrosis [28, 29]. Clinical evidence showed that more neutrophils and higher neutrophil elastase, MPO, and collagenase levels could be observed in IPF patients [30, 31]. More neutrophil infiltration and MPO staining were detected in the bleomycin-treated rodents based on the histological study [32]. Our study showed that more MPO staining was observed in WT BLM group mice, which could be concluded that 5-HT might promote neutrophil infiltration and MPO release. Duerschmied et al. discovered that 5-HT could promote the recruitment of neutrophils to sites of acute inflammation in mice [33]. Jang et al. also found that 5-HT could promote MPO expression in mouse liver from cholestatic injury induced by bile duct ligation [34]. Our previous study also proved that 5-HT could promote neutrophil infiltration and MPO release in the lung and liver tissues affected by abdominal sepsis [35].

Pulmonary fibrosis is thought to be a chronic inflammatory disease of the lung parenchyma. Histological analysis results show that massive inflammatory cells, including lymphocytes, macrophages, and neutrophils, accumulate in pulmonary fibrosis-affected lung tissues. In addition, high serum and BALF inflammation-related cytokines are observed in rodents and IPF patients [23]. TNF-*α* is a powerful proinflammatory cytokine that promotes the infiltration of inflammatory cells and the proliferation of fibroblasts. IL-6 is a downstream cytokine that may modulate pulmonary inflammation and fibrosis directly. Increased TNF-*α* and IL-6 levels in serum and BALF are associated with lung fibrosis [36]. In our study, we found that BALF TNF-*α* and IL-6 levels and lung TNF-*α* and IL-6 mRNA were markedly increased in the WT BLM group mice, indicating that 5-HT might promote the production of cytokines in pulmonary fibrosis. The differences in the inflammatory components between WT BLM and KO BLM groups were even more evident than fibrosis scores. The reason was that inflammatory reaction played a key role in the development of pulmonary fibrosis, and 5-HT could affect the fibrosis denouement through the inflammation process. The role of 5-HT in the modulation of inflammation and the immune system can be acquired from the review written by Shajib and Khan [8]. In our previous studies, we also found that 5-HT could increase the production of TNF-*α* and IL-6 in MODS, sepsis, and PPA formation [13, 35, 37]. Moreover, our results also showed that 5-HT might aggravate the toxic effect of bleomycin on the capillary endothelium and alveolar epithelium and thus promote exudation of proteins and cells which were reflected by the high BALF proteins, cells, and cytokine levels. We speculated that 5-HT might promote the exudation of proteins and cells through bleomycin-induced inflammation reactions in the lung could contribute pulmonary fibrosis finally.

Imbalance between the excessive generation of ROS and the disability of antioxidant causes oxidative stress, which plays a major role in pulmonary fibrosis. Remarkable elevation of oxidant burden and disability of antioxidant are observed in lung fibrosis [24]. In this study, we demonstrated that 5-HT did affect the antioxidant/oxidant balance and exacerbate oxidative stress in bleomycin-induced lung fibrosis. The role of 5-HT in oxidative stress has been well studied. Nocito et al. found that 5-HT mediated oxidative stress and mitochondrial toxicity in a murine model of nonalcoholic steatohepatitis [38]. Our previous data showed that 5-HT could increase the production of ROS in septic lung and liver tissues and decrease the SOD and GSH levels in PPA tissues [13, 35].

In conclusion, our preclinical study using TPH1 knockout and wild-type C57BL/6 mice demonstrated that 5-HT markedly exacerbated bleomycin-induced lung fibrosis. The potential mechanisms might be that 5-HT could facilitate collagen deposition, inflammation, and oxidative stress during pulmonary fibrosis. These findings indicated that 5-HT could be a biomarker of pulmonary fibrosis and might be a therapeutic target in the future.

## Figures and Tables

**Table 1:** The inflammatory score system.

Score	Description
0	No infiltration of inflammatory cells
1	Occasionally by vein and bronchus cuff inflammatory cells infiltration
2	The majority of vein, peribronchial infiltration of inflammatory cells, inflammatory cell layer 1~5
3	The majority of vein, peribronchial infiltration of inflammatory cells, inflammatory cell layer is greater than 5

**Table 2:** The fibrotic score system.

Score	Description
0	No pulmonary fibrosis
1	Mild pulmonary fibrosis, the affected area was less than 20%
2	Moderately pulmonary fibrosis, involvement of area of 20%~50%
3	Severe pulmonary fibrosis, the affected area was more than 50%, the alveolar structure disorder

**Table 3:** The Ashcroft fibrosis score system.

Score	Description
0	Normal lung; no fibrotic burden at the flimsiest small fibers in some alveolar walls
1	Lung structure: alveoli partly enlarged and rarefied, but no fibrotic masses was present; alveolar septa: isolated gentle fibrotic changes (septum ≤ 3× thicker than normal)
2	Lung structure: alveoli partly enlarged and rarefied, but no fibrotic masses; alveolar septa: clearly fibrotic changes (septum > 3× thicker than normal) with knot-like formation but not connected to each other
3	Lung structure: alveoli partly enlarged and rarefied, but no fibrotic masses; alveolar septa: contiguous fibrotic walls (septum > 3× thicker than normal) predominantly in the whole microscopic field
4	Lung structure: single fibrotic masses (≤10% of microscopic field); alveolar septa: variable
5	Lung structure: confluent fibrotic masses (>10% and ≤50% of microscopic field), lung structure severely damaged but still preserved; alveolar septa: variable
6	Lung structure: large contiguous fibrotic masses (>50% of microscopic field), lung architecture mostly not preserved; alveolar septa: variable, mostly not exist
7	Lung structure: alveoli nearly obliterated with fibrous masses but still up to five air bubbles; alveolar septa: nonexistent
8	Lung structure: alveoli nearly obliterated with fibrous masses but still up to five air bubbles; alveolar septa: nonexistent
